# Siderite and vivianite as energy sources for the extreme acidophilic bacterium *Acidithiobacillus ferrooxidans* in the context of mars habitability

**DOI:** 10.1038/s41598-024-64246-7

**Published:** 2024-06-27

**Authors:** Gabriel Gonçalves Silva, Roberta Almeida Vincenzi, Gabriel Guarany de Araujo, Sara Jéssica Soja Venceslau, Fabio Rodrigues

**Affiliations:** 1 Programa de Pós-Graduação Em Química, Institute of Chemistry, University of São Paulo, São Paulo, Brazil; 2 Programa de Pós-Graduação Em Bioquímica E Biologia Molecular, Institute of Chemistry, University of São Paulo, São Paulo, Brazil; 3 Department of Microbiology, Institute of Biomedical Sciences, University of São Paulo, São Paulo, Brazil; 4 Institute of Biosciences, University of São Paulo, São Paulo, Brazil; 5 Departamento de Química Fundamental, Institute of Chemistry, University of São Paulo, São Paulo, Brazil

**Keywords:** Siderite, Vivianite, *Acidithiobacillus ferrooxidans*, Mars, Astrobiology, Biogeochemistry, Astrobiology

## Abstract

Past and present habitability of Mars have been intensely studied in the context of the search for signals of life. Despite the harsh conditions observed today on the planet, some ancient Mars environments could have harbored specific characteristics able to mitigate several challenges for the development of microbial life. In such environments, Fe^2+^ minerals like siderite (already identified on Mars), and vivianite (proposed, but not confirmed) could sustain a chemolithoautotrophic community. In this study, we investigate the ability of the acidophilic iron-oxidizing chemolithoautotrophic bacterium *Acidithiobacillus ferrooxidans* to use these minerals as its sole energy source. *A. ferrooxidans* was grown in media containing siderite or vivianite under different conditions and compared to abiotic controls. Our experiments demonstrated that this microorganism was able to grow, obtaining its energy from the oxidation of Fe^2+^ that came from the solubilization of these minerals under low pH. Additionally, in sealed flasks without CO_2_, *A. ferrooxidans* was able to fix carbon directly from the carbonate ion released from siderite for biomass production, indicating that it could be able to colonize subsurface environments with little or no contact with an atmosphere. These previously unexplored abilities broaden our knowledge on the variety of minerals able to sustain life. In the context of astrobiology, this expands the list of geomicrobiological processes that should be taken into account when considering the habitability of environments beyond Earth, and opens for investigation the possible biological traces left on these substrates as biosignatures.

## Introduction

Recently, the knowledge about the planetary environments in our neighborhood has been vastly increased with advances in space research and exploration. Particularly, Mars has been intensely studied, demonstrating that in its past it had similar conditions to those found on Archaean/Proterozoic Earth^1^. Although present Mars presents evidence of possible underground liquid water and transient surface water, its environment presents several challenges for the establishment of life as we know it, such as low concentrations of organic matter, a high index of ultraviolet radiation, high concentrations of oxidizing salts, low atmospheric pressure, among many other deleterious conditions^2^^,^^3^. Nevertheless, the formation of some microenvironments with specific characteristics can mitigate some challenges for the development of life as we know it.

Near the South Pole of the planet, evidence of subsurface lakes has been observed, harboring liquid water thanks to a high concentration of dissolved salts^4^. Although these subsurface environments prohibit the development of photosynthesizing organisms, life under these conditions would be protected against ultraviolet radiation from the Sun and also, to some degree, from more energetic radiation that comes from space as cosmic rays^5^.

Further exploration of the planet has revealed that Mars presents numerous minerals that are formed in the presence of water, like clay minerals, carbonates and sulfates, iron oxides, among others^6^. Geological features that corroborate the past presence of widespread liquid water (*e.g.* abandoned deltas, fans, channels, and exhumed channels) can also be observed on the surface of the planet^7^. Such aqueous environments probably presented distinct chemical compositions derived from their geochemical contexts and could range, for example, from alkaline to acidic waters^8^. This range of conditions would lead to mineral diversification, which can then be studied to assess the habitability of past and present Mars environments.

Today, the harsh conditions on the surface of Mars are not favorable for the preservation of significant amounts of organic matter, though a more widespread presence of it in the past cannot be discarded^9^. Chemolithoautotrophy is regarded as a possible microbial metabolism able to fill niches where water could be available on Mars, as it allows for the consumption of inorganic compounds, possibly the main energy sources available for microbial growth on the planet^10^. This form of metabolism is observed in analog environments on Earth^11^ and has been explored for applications in energy production^12^, industrial scale processing of ores^13^, and even in space exploration, for example, in resource recovery and energy production on Mars^14^. Studies have evaluated the microbe-mineral interactions between different organisms and rocks in the space environment, such as in the BioRock experiment at the International Space Station^15,16^. More specifically, there is also growing interest in studies using meteorites, relatively easily accessible materials representative of rocks from different origins in the Solar System^17^, in addition to simulated Martian regolith as substrates for these mineral-microorganism interaction studies^18^.

Considering only chemolithoautotrophic organisms interacting with meteorites, a pioneering work evaluated a metallic meteorite as an energy source to sustain the growth of the extreme acidophilic bacteria *Leptospirillum ferrooxidans* and *Acidithiobacillus ferrooxidans*^19^. Considering its status as a model organism for bacterial iron oxidation, later studies also used *A. ferrooxidans* to access the capacity of other substrates to sustain its growth, like carbonaceous chondrites and metallic meteorites^20^. Nevertheless, it is important to note that other chemolithoautotrophic organisms have also been explored in this context, such as the archaeon *Metallosphaera sedula* with ordinary chondrites^21^ and Martian meteorites^22^ as substrates.

*A. ferrooxidans* is a Gram-negative extremophilic bacterium that displays a broad metabolic versatility, capable of living in aerobic and anaerobic conditions and oxidizing different species of reduced sulfur and iron, growing optimally in pH 1.8 to 2.0. Industrially, it is of great importance in biomining and bioleaching processes of copper ores^23,24^. The oxidation of high concentrations of iron (as Fe^2+^) during its growth is associated with the precipitation of minerals including Fe^3+^ oxyhydroxides and oxyhydroxide sulfates. In *A. ferrooxidans* cultures, the first mineral to precipitate is schwertmannite (Fe^3+^_8_O_8_(OH)_8−2x_(SO_4_)_x_ ⋅nH_2_O, x = 0.5 to 2), a metastable low crystallinity phase^25^, that gradually reacts to form jarosite (XFe^3+^_3_(SO_4_)_2_(OH)_6_, with X representing, usually, a monovalent cation such as K^+^, NH_4_^+^, H_3_O^+^, etc.)^26^.

Although the formation of jarosite can be biotically mediated, it can also happen abiotically in natural environments. Even if these minerals have been proposed as possible biosignatures since isotopic fractionation in iron oxyhydroxides has been associated with biological activity, its unambiguous identification as such and the correct differentiation between its biotic and abiotic origin are still difficult to achieve^20^. This is important since large areas showing signals of jarosite were reported on Mars, related to paleoenvironments with acidic waters^27^.

In this context, the importance of studying iron-oxidizing organisms in a Martian environment arises^6^. As an example, Bauermeister^28^ tested different kinds of simulated Martian regolith as iron sources for the growth of *A. ferrooxidans* in combination with other conditions present in the planet, such as UV-C radiation, salts, and low temperatures. The simulated regolith used in this study contained Fe-bearing minerals such as olivine, hematite, chamosite, goethite, and siderite. However, no studies were conducted to evaluate the capacity of each specific mineral to sustain the growth of this organism.

Siderite (Fe^2+^CO_3_) has been detected on the surface of Mars by the Phoenix lander^29^ and also on Mars-derived SNC meteorites^30,31^. Meanwhile, on Earth, siderite stands out as one of the main Fe^2+^-bearing minerals and can be oxidized by autotrophic microorganisms^32^. This mineral is associated with biogeochemical cycles and is found in different contexts, such as past depositions of banded iron formations (BIF) or marine sedimentation zones, occurring in connection with potential biosignatures^33^, Vuillemin et al.^34^.

Vivianite (Fe^2+^_3_(PO_4_)_2_.8H_2_O) is another mineral that can be associated with biogeochemical processes. The mineral is found in freshwater and marine systems as a biogenic product^35^ while it can undergo oxidation caused by the action of neutrophilic^36^ or photoautotrophic microorganisms^37^. On Mars, even though no positive detection of vivianite has been done, its presence, along with other phosphates, can be expected in patches with high concentration of phosphorus, since other common minerals could mask their existence^38^. Siderite and vivianite, containing reduced iron and presenting high solubility in acidic solutions can be targets for acidophilic iron-oxidizing bacteria like *A. ferrooxidans* but this interaction was still unexplored.

In this study, we describe the capacity of *A. ferrooxidans* to utilize siderite and vivianite as its sole energy source for growth in the complete absence of organic matter. The ability of this extremophile to use Fe^2+^ from these minerals as an electron donor for microbial metabolism was explored in the context of Martian habitability. In addition, we report the novel finding that this organism can fix carbon directly from the siderite carbonate for biomass production, expanding the habitable environments of Mars to include places with little or no contact with its atmosphere.

## Material and methods

### Bacterial strain and culture media

The experiments were carried out using *A. ferrooxidans* strain LR, isolated from an acid leachate of a uranium mine at Lagoa Real, in the state of Bahia, Brazil^39^. Subcultures of *A. ferrooxidans* were routinely kept in the laboratory at 30 °C in modified T&K medium^40^, made by the mixing of two solutions, named A and B. Solution A contains (NH_4_)_2_SO_4_ (0.625 g/L), K_2_HPO_4_ (0.625 g/L) and MgSO_4_·7H_2_O (0.625 g/L), with the addition of concentrated H_2_SO_4_ to reach pH 1.8. Solution A is sterilized by autoclaving at 121 °C. Solution B contains FeSO_4_·7H_2_O (166.7 g/L) with the addition of concentrated H_2_SO_4_ to reach pH 1.8 and is sterilized by filtration. The culture medium is a mixture of solutions A and B in a 4:1 ratio, resulting in a final solution with 120 mM of Fe^2+^.

For the experiments using minerals (siderite or vivianite) as the sole iron source, T&K medium was modified by removing the soluble iron (FeSO_4_·7H_2_O) and supplementing it with the powdered mineral (< 125 μm particle size). These media are henceforth designated siderite medium and vivianite medium.

During the flask experiments without atmospheric CO_2_, a culture medium with both soluble iron (FeSO_4_·7H_2_O) and siderite was also used, designated siderite-FeSO_4_ medium. In those cases, the mass of powdered mineral added to the culture medium was calculated so the concentration of the total Fe^2+^/CO_3_^–2^ would be approximately 5 mM. This value was chosen so that the mineral dissolution would have little effect on the overall pH during the experiment. The final powdered mineral mass used in each case was calculated using the mass of the pure form of the mineral and took into account the degree of purity obtained from the characterizations of the substrates (Section "Mineral characterization".). During the sealed flask experiments with inert gas, T&K medium with 60 mM of soluble iron (FeSO_4_·7H_2_O) was inoculated (as described in Section "Stock and inoculum cultures".) and was grown for 48 h. After all the Fe^2+^ was oxidized to Fe^3+^, 10 mL of the medium was transferred to the glass vial, 10 g/L of sulfur (S_8_) was added and the glass vial was crimped, being then designated Fe^3+^-sulfur medium. In one of the conditions, approximately 5 mM of siderite (like previously explained) was also added.

### Culturing procedures

#### Stock and inoculum cultures

For each experiment, an inoculum culture was prepared using 50 mL sterile Erlenmeyer flasks with cotton plugs containing 20 mL of modified T&K medium, inoculated with 2% (v/v) of the stock cell suspension of *A. ferrooxidans*, and grown at 30 °C in an incubator shaker table at 180 rpm. The Fe^2+^ oxidation to Fe^3+^ in the inoculum culture was monitored using the colorimetric method (described in Section "Colorimetric quantification of Fe^2+^ and Fe^3+^".) to identify the exponential growth phase (40 to 80 mM Fe^3+^). Only during this phase the inoculum culture was used as a source to inoculate the experiments.

#### Bioreactor experiments with siderite

A bioreactor system was assembled with two 100 mL stirred-tanks (working volume) operated as two parallel batches in similar conditions (duplicates for each individual run). Experiments were conducted to assess two distinct conditions: an abiotic assay (to evaluate the abiotic siderite dissolution and oxidation of Fe^2+^) and a biotic assay (inoculated with 2% (v/v) of an *A. ferrooxidans* inoculum culture at the exponential growth phase). In each experiment, the previously sterilized vessels started with 100 mL of siderite medium, maintained at 30 °C with a vigorous stir, constant aeration, and pH control (within the 1.7–1.9 range). The system was kept running for one week with daily sampling. The system, whose layout is presented in the Online Resource 1, is composed by an air pump (nominal air flow of 2 L/min) bubbling the air through a packed bed water column (1–2 mm glass spheres in a 250 mL Dreschel bottle) for humidity saturation, designed to reduce water drag from the medium. The air that leaves the Dreschel bottle reaches a splitter with a small valve control that separates the two air flows, one for each bioreactor. Each flow goes through a tube with a lateral port used to manually inject small amounts of pH = 0 H_2_SO_4_ solution to correct the pH of the culture during the experiment. Before reaching the bioreactor, the flow crosses a sterilizing filter to avoid any medium contamination by the air. Each bioreactor is arranged in a 100 mL Schott bottle whose cap was cut at the top and fitted with a rubber septum traversed by four elements: inlet tube for aeration (water-saturated and filtered air), outlet sampling tube coupled with a needleless syringe, pH sensor, and air outlet (hypodermic needle covered with hydrophobic cotton). Each bioreactor is placed inside a beaker filled with water, monitored by a thermocouple from the thermostatic magnetic stirrer that controls the temperature and the stir bar inside the bioreactor.

#### Sealed vial experiments without atmospheric CO_2_ with siderite

For this experiment, each culture was made inside a sterilized 30 ml crimped glass vial, containing 10 ml of culture medium and, for the biotic assays, 2% (v/v) of an *A. ferrooxidans* inoculum culture in the exponential growth phase. A holdup gas exchange system was assembled with two hypodermic needles punching through the rubber septum, one of them connected to a pressurized gas line of synthetic air (80% N_2_ and 20% O_2_, White Martins, 99.995% of purity), to bubble CO_2_-free gas inside the vial, and the other hypodermic needle to purge the outlet gas. The system was kept running for several minutes in each vial until all the atmospheric CO_2_-rich air was expelled from the flask. Six conditions were assembled (each one in triplicate): abiotic and biotic vials with modified T&K medium; abiotic and biotic vials with siderite medium; abiotic and biotic vials with siderite-FeSO_4_ medium (Section "Bacterial strain and culture media".). All 18 vials were then conditioned at 30 °C in a shaker table at 180 rpm during one week, with two sample withdraws: before crimping at the beginning and after the opening at the end.

#### Sealed vial experiments with siderite and sulfur in an inert atmosphere

This experiment was assembled similarly to the flask experiments without atmospheric CO_2_ with siderite (Section "Sealed vial experiments without atmospheric CO_2_ with siderite"), however the pressurized gas used was pure helium (CO_2_-free and O_2_-free, White Martins, 99.995% purity). Two conditions were made in triplicate: Fe^3+^-sulfur medium with and without the addition of approximately 5 mM of siderite (as explained in Section "Bacterial strain and culture media".). Prior to the flask closing, no new inoculum was added, as the oxidation of the Fe^2+^ was already carried out by the initial addition of *A. ferrooxidans* to prepare the Fe^3+^-sulfur medium. Again, all 6 vials were then conditioned at 30 °C in a shaker table at 180 rpm during one week, with two sample withdraws: before crimping at the beginning and after the opening at the end.

#### Experiments with baffled Erlenmeyer flasks and vivianite

The vivianite growth assays were performed using sterile 50 ml baffled Erlenmeyer flasks with a hydrophobic cotton plug containing 20 ml of vivianite medium and, for the biotic assays, 2% (v/v) of an *A. ferrooxidans* inoculum culture in the exponential growth phase. Both abiotic and biotic conditions were performed in triplicate. After inoculations, the baffled Erlenmeyer flasks were conditioned at 30 °C in a shaker table at 180 rpm during one week, with daily sampling.

### Culture analytical procedures

#### Colorimetric quantification of Fe^2+^ and Fe^3+^

To estimate the presence of the Fe^2+^ and Fe^3+^ species in solution and in the minerals, colorimetric quantification of both ions were continuously employed according to the methodology described in Gonçalves Silva et al.^23^. Complexation of the ferric ion (Fe^3+^) was performed with a solution of potassium thiocyanate acidified with sulfuric acid, while the ferrous ion (Fe^2+^) was complexed with the solution of *o*-phenanthroline buffered with sodium acetate as described by Mendham et al.^41^. Complexes of Fe^2+^ and Fe^3+^ were measured at the wavelengths of 510 and 470 nm, respectively, in a 10 mm-pathlength cuvette using an UV–Vis spectrophotometer (Thermo Scientific NanoDrop 2000c). For quantification of Fe^2+^ and Fe^3+^ in the mineral phase, an aliquot taken from the experiment was centrifuged, the supernatant was discarded, and the pellet was washed with an equal volume of a solution of H_2_SO_4_ 0.1 M. The process was repeated once more, and the final pellet was solubilized with an equal volume of a solution of HCl 1 M.

#### Most probable number (MPN) determination of culturable cells

The determination of the concentration of culturable cells was performed based on the modified Most Probable Number (MPN) assay described by Bauermeister et al.^42^. An aliquot of culture medium containing the *A. ferrooxidans* taken from each flask of an assay underwent an 8-step serial dilution in modified T&K medium, in which each step is 10 × more diluted than the previous one (corresponding to dilutions from 10^1^ to 10^8^). After the serial dilution, 20 μl of each dilution was transferred to separate wells of a 96-wells microplate containing 180 μl of modified T&K medium (process performed in six replicates). The microplate was then transferred to a bacteriological incubator without shaking at 30 °C for growth for 14 to 21 days. The wells in which the *A. ferrooxidans* growth was observed (identified by the distinctive appearance of the orange-like color in the solution associated with the Fe^3+^ formation) were enumerated and the Most Probable Number (MPN) was determined using a calculation routine called MPN calculation program (Version 6)^43^. For the bioreactor experiments, the MPN was determined in every sampling performed, while for both flask experiments only the initial and final MPN were obtained.

### Mineral characterization

The minerals used in the experiments were bought from experienced mineral dealers. The vivianite sample, a mineral with theoretical formula Fe^2+^_3_(PO_4_)_2_·8H_2_O, was from Bolivia. The siderite sample was from Morro Velho mine, Nova Lima, Minas Gerais, Brazil. Both mineral samples were ground in a ceramic mortar and pestle until forming a fine powder that was then sifted through a 120 Mesh sieve, allowing the collection of the mineral particulate fraction under 125 μm of diameter. The procedures for characterization of the minerals are described as following (except for the Fe^2+^ and Fe^3+^ quantification that has already been described in the Section "Colorimetric quantification of Fe^2+^ and Fe^3+^".).

The theoretical formula of siderite is Fe^2+^CO_3_, however this mineral is rarely found as a pure specimen, usually having some amount of magnesium in its composition, taking part of a mineral series called the Magnesite-Siderite series. The chemical characterization of the siderite used in the experiments allowed the calculation of its chemical formula as (Mg_0.563_Fe_0.422_Mn_0.008_Ca_0.006_)CO_3_. This result puts the sample in the ferroan-magnesite range of the Magnesite-Siderite series, also known as “mesitite”^44^, being characterized as a solid solution mineral with Mg:Fe ratio between 70:30 and 50:50. As the focus of the experiments is in the interaction between the microorganism and the Fe^2+^ phase of the minerals, the ferroan-magnesite used in the experiments is referred just as siderite in this work. The ground siderite also showed a 3.9% (w/w) of insoluble phase, giving a total recovery of 97.1% (w/w) of the analyzed sample. The result of the specific surface area measurement was 6.6873 m^2^/g for the siderite powder used for the experiments.

Vivianite is a very light-sensitive mineral with an ideal composition of Fe^2^^+^ _3_(PO_4_)_2_·8H_2_O. However, in normal conditions, it slowly oxidizes, changing its composition accordingly to the ideal formula: Fe^2^^+^_3-x_Fe^3+^_x_(PO_4_)_2_(OH)_x_·(8-x)H_2_O, where 0 ≤ x ≤ 2, until reaching the composition of the mineral called metavivianite, whose ideal formula is Fe^2^^+^ Fe^3^^+^ _2_(PO_4_)_2_(OH)_2_·6H_2_O^45^. The chemical formula estimated for the mineral at the moment it was used in the experiments, although slightly different from the ideal, was determined as Fe^2^^+^ _2.202_Fe^3^^+^ _0.798_(PO_4_)_2_(OH)_0.798_·7.063H_2_O. The result of the specific surface area measurement was 5.1411 m^2^/g for the powder used for the experiments.

#### Inductively coupled plasma optical emission spectrometry (ICP-OES).

The mineral powders were solubilized in a solution of 1 M of HCl, diluted in MilliQ water and characterized by the methodology described in Gonçalves Silva et al.^23^. The ICP OES analyses were carried out in iCAP 6300 Duo ICP optical emission spectrometer (Thermo Fisher Scientific) equipped with axial and radial viewed plasma and a charge injection device detector, which allows measurements from 166.25 to 847.00 nm. An echelle polychromator was purged with argon, and a radiofrequency source of 27.12 MHz was used. The ICP OES was operated in a radiofrequency power of 1250 W using a Meinhard nebulizer, a cyclonic spray chamber and an axial torch orientation. The plasma gas flow was 15 L/min , the intermediate gas flow was 1 L/min and the nebulizer gas flow was 0.45 L/ min . The samples were introduced at 1.5 mL /min and the wavelengths used for the identification of the elements were: 317.9 nm for Ca, 238.2 nm for Fe, 285.2 nm for Mg and 257.6 nm for Mn.

#### Carbonate quantification

Carbonate concentration was measured by back titration. A weighted sample of siderite powder was slowly solubilized in a solution of known volume of HCl (1 M), with the release of the CO_3_^2+^ as CO_2_ and the consumption of 2 H^+^. After all siderite was solubilized, a titration was performed in the final solution using a solution of 1 M of NaOH in the presence of a pH meter. The amount of NaOH used to neutralize the remaining acid in solution allowed calculating the HCl spent in the siderite dissolution and, by stoichiometry, calculating the original amount of carbonate in the sample.

#### Phosphate quantification

A weighted sample of vivianite powder was solubilized in a known volume of HCl (1 M) and later diluted in demineralized water to perform the colorimetric quantification of PO_4_^3+^ ions according to the modified methodology described in Lozano-Calero et al.^46^. A reaction solution was prepared with sulfuric acid (15%), antimony potassium tartrate (4.5 mM), ammonium heptamolybdate (10 mM) and ascorbic acid (100 mM), added, in this order, in the proportion of 5:1:5:5. The reaction was performed adding the sample and the reaction solution in the proportion of 3:2 and incubated for 30 min. The complex formed was measured at 830 nm using an UV–Vis spectrophotometer.

#### Insoluble phase quantification

A weighted sample of mineral powder was slowly solubilized in a known volume of HCl (1 M). The solution was then filtered in a previously weighed dry filter paper of 0.45 μm pores. The filter paper was dried in a drying oven at 60 °C for 48 h and was weighted again. The difference in the weight was used to obtain the amount of minerals in the powder that are insoluble in the acidic solutions.

#### Specific surface areas

The specific surface areas were obtained for both mineral powders in a Micromeritrics ASAP 2020 surface area analyzer by the Brunauer–Emmett–Teller (BET) nitrogen adsorption method. The vivianite sample needed to be previously dried by being heated to 120 °C overnight to avoid dehydration during the procedure, based on the methodology by Luna-Zaragoza et al.^47^. The samples were degassed and analyzed using a multipoint nitrogen adsorption/desorption routine at room temperature.

#### Hydration quantification

The determination of the total number of hydration water molecules in vivianite was performed by thermogravimetric analysis (TG-MS), using a TG-DSC Netzsch thermobalance, model STA 409 PC Luxx. A small sample of the powdered mineral was placed into the alumina crucible of the equipment and a linear heating rate of 10 °C/min was applied, raising the temperature to 1000 °C, meanwhile, a synthetic air flow of 50 mL/min was maintained. The outflow was coupled to a QMS 403C Aeolos mass spectrometer set up to detect the presence of molecules with the water 18 m/z signal.

### Modelling

#### Dissolution kinetics

The dissolution process of the minerals in the presence or absence of *A. ferrooxidans* was interpreted as similar to the kinetics of a first-order reaction. The direct quantification of the mineral concentration as a solid phase in the culture medium faces some difficulties: due to the production of EPS by the cells^48^, there is a small loss of mineral that ultimately adheres to the bioreactor walls,besides that, the non-homogeneity of the solid phase inside the bioreactor can also introduce bigger errors to the analysis. To avoid such errors, it was opted to use the Fe^2+^ concentration (product of the mineral dissolution in the abiotic experiments) as the input for the first-order model fitting. In the biotic experiments, the total Fe^3+^ concentration minus the Fe^3+^ concentration present in the mineral (product of the Fe^2+^ biological oxidation) was used as input, as the dissolution process is considered the limiting step. The model fitting was performed using the Eq. (1)^49^

1 $$c = c_{s} *(1 - exp\left( { - k*t} \right))$$

where c is the concentration of the product of the dissolution reaction (Fe^2+^ in the abiotic assays and Fe^3+^ in the biotic assays) [mM/L]; c_s_ is the final concentration (in t → infinite) of the product of the dissolution reaction [mM/L]; k is the dissolution rate [h^−1^]; and t is time [h].

#### Growth kinetics

The microorganism growth rate was directly estimated by fitting a growth kinetics model on the cell concentration estimate obtained by the MPN method (described in Section "Most probable number (MPN) determination of culturable cells".). In the absence of cell concentration data, an indirect growth rate value was obtained using the dissolution kinetics (as described in Section "Dissolution kinetics"). This approximation is possible because it was demonstrated that the dissolution process was the limiting phase during the *A. ferrooxidans* growth. The growth kinetics model used is shown in Eq. 2^50^.

2 $$X = X_{0} *(exp\left( {\mu_{net} *t} \right))$$

where X is the cell concentration in the exponential phase [MPN/L]; X_0_ is the initial cell concentration in the exponential phase [MPN/L]; μ_net_ is the growth rate [h^−1^]; and t is time [h].

## Results

### Siderite cultures

#### Bioreactor experiments

**Figure 1:**
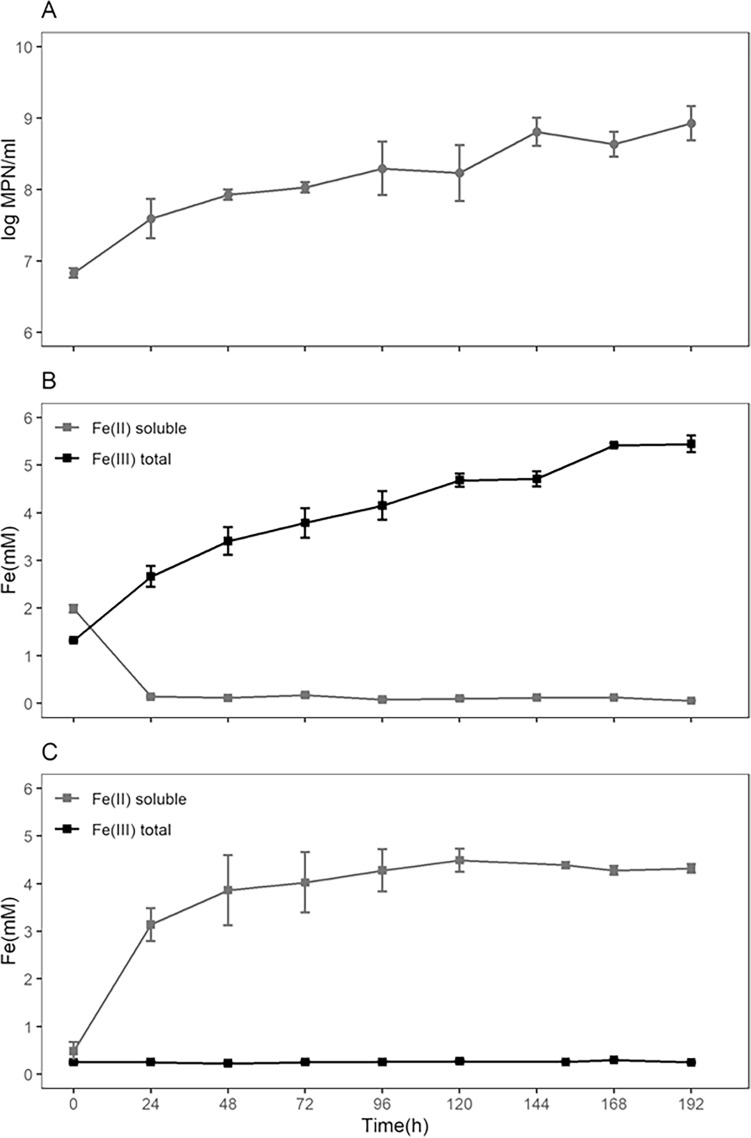
Bioreactor experiments using siderite. (**a**) Increase in the culturable cell concentration (MPN) during the biotic assay (coinciding with the Fe^2+^ oxidation, Spearman correlation ρ = 0.967 for *p* > 0.001). (**b**) Biotic assay showing the microorganism-mediated soluble Fe^2+^ oxidation and total Fe^3+^ accumulation. (**c**) Abiotic assay showing the siderite dissolution with Fe^2+^ accumulation and very low oxidation to Fe^3+^.

The siderite abiotic assay was performed using bioreactors operating as two parallel batches in similar conditions (duplicates). This assay allowed the evaluation of the dissolution of the siderite in the culture medium and the natural process of oxidation of the Fe^2+^ ion while optimum culture conditions were kept (30 °C, constant aeration and pH control within the 1.7–1.9 range). After 192 h of experiment, the dissolution of the siderite was carried out until almost completeness (Fig. 1c).

**Figure 2:**
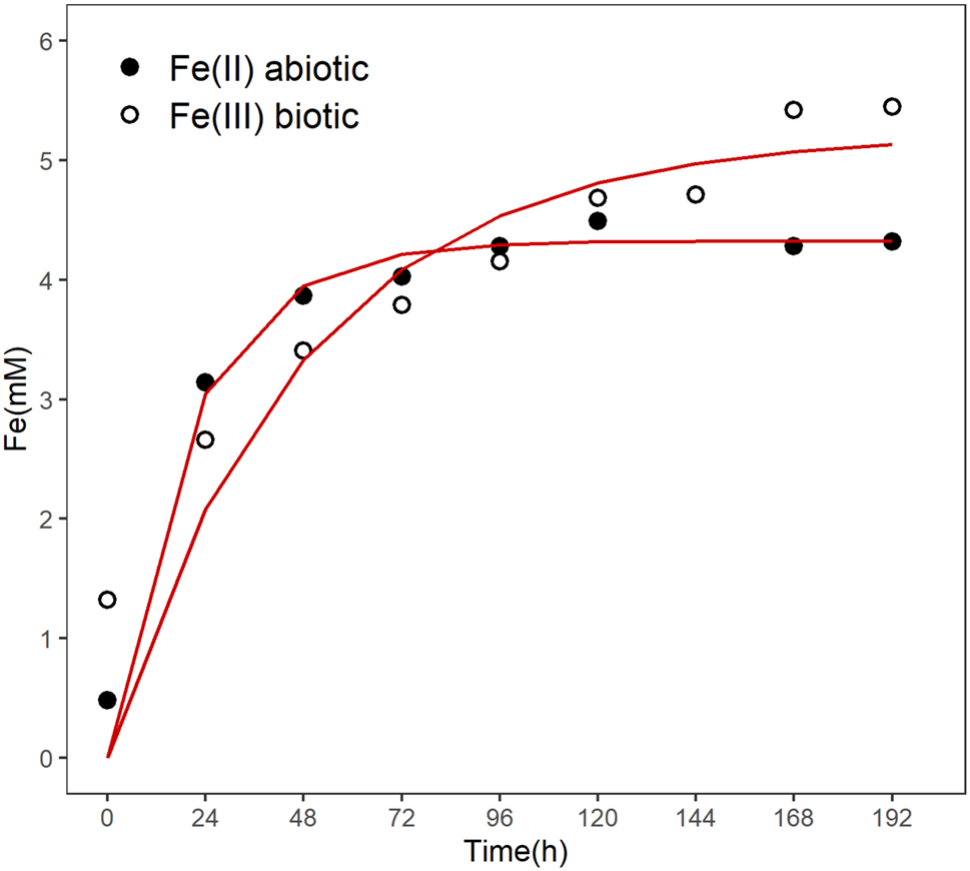
First-order model fitting for the siderite dissolution during the bioreactor experiments by the direct accumulation of Fe^2+^ (abiotic assay) and accumulation of Fe^3+^ after the biotic oxidation of Fe^2+^ to Fe^3+^ (biotic assay) (see Section “Modelling”).

It was possible to see that, after 120 h of the experiment, the concentration of minerals in the solid phase dropped to almost zero, observed as a color change of the medium: going from an opaque cream-white suspension to a light white and transparent medium with very low turbidity. The Fe^3+^ concentration was constant and near zero for the duration of the abiotic experiment, indicating low abiotic oxidation. The Fe^2+^ concentration grew faster in the first 48 h, and then slowed down until reaching a constant value in the last samplings, indicating the stabilization of siderite solubilization. This pattern was interpreted as similar to the kinetics of a first-order reaction, and the fitting of such model gave a dissolution rate of k = 0.05 ± 0.01 h^−1^ (Fig. 2). Considering the specific surface area, this represents 9.22*10^–9^ mol/(s*m^2^) for pure siderite, that is similar to both values (9.98*10^–9^ and 1.15*10^–8^ mol/(s*m^2^)) obtained by Cullen et al.^51^, in similar conditions (pH = 2 and 0.01 M of MgSO_4_). A summary of the results can be found in Table 1.

**Table 1:** Summary of the dissolution and growth rates obtained in the abiotic and biotic cultures using siderite and vivianite.

Mineral		Dissolution rate (h^−1^)*	Specific dissolution rate (mol/s*m^2^)	Growth rate (h^−1^)*
Siderite	Abiotic	0.05 ± 0.01	9.22*10^–9^	–
	Biotic	0.021 ± 0.006	3.87*10^–9^	0.017 ± 0.002
Vivianite	Abiotic	0.027 ± 0.011	2.93*10^–9^	–
	Biotic	0.033 ± 0.014	3.85*10^–9^	0.07 ± 0.03

*The confidence interval corresponds to the parameter fitting error.

The biotic assay was performed in the same conditions as the abiotic assay, but it was inoculated with 2% (v/v) of the stock cell suspension of *A. ferrooxidans* at the exponential growth phase. The inoculum introduces in the system a small amount of Fe^2+^ and Fe^3+^ from the previous culture and can be seen in the first sample point (Fig. 1b) (for the calculation of the dissolution kinetics, these values were not taken into account). The initial Fe^2+^ is consumed prior to the next sampling (24 h) and during the rest of the experiment is always negligible, demonstrating that the biological oxidation was the faster step while the siderite dissolution was the limiting step of the process. These facts make possible the use of the total Fe^3+^ concentration (soluble and precipitated Fe^3+^) for the kinetic modelling (Section "Dissolution kinetics".).

Cultures using T&K medium with only soluble Fe^2+^ show the precipitation of Fe^3+^ minerals (characterized by its yellow color) after reaching Fe^3+^ concentrations up to 100 mM^23^. In the cultures described in the present work, using the siderite medium, the observation of a slow color change began even before the first 24 h period. The culture went from opaque cream-white at the beginning to a light beige/yellowish suspension with a higher turbidity than the observed at the end of the abiotic assay, indicating the formation of a precipitate. Besides that, there was a visible and increasing deposition of the precipitate, made up of a mixture of the minerals (siderite and Fe^3+^ minerals) in the walls of the bioreactor, making it difficult to execute the quantification with high precision.

The total Fe^3+^ showed a slow increase during the whole experiment, reaching a probable stability only in the last two samplings, while not all of the siderite was dissolved. To confirm that the oxidation was being used by *A. ferrooxidans* cells to obtain energy, the MPN methodology was performed to determine the concentration of culturable cells during the experiment. The cell concentration during the experiment also showed an increase, reaching two orders of magnitude of growth (Fig. 1a). The MPN concentration growth coincided with the Fe^3+^ curve pattern (Spearman correlation ρ = 0.967, *p* > 0.001). The dissolution kinetics for the siderite in the biotic assay was found to be much slower than the one observed for the abiotic assay (Fig. 2), demonstrating how the action of this microorganism can strongly influence the mineral dissolution. Although the process behind this difference is not yet understood, the production of EPS by the bacteria could be responsible for covering the mineral and reducing the contact area between the mineral and the solution. The dissolution rate obtained was k = 0.021 ± 0.006 h^−1^, this represents 3.87*10^–9^ mol/(s*m^2^), that is, more than twofold slower than the abiotic assay. The pattern of growth rate obtained for the *A. ferrooxidans* using the MPN assay shows a fast increase of the cellular concentration in the first 24 h, when the Fe^2+^ available was abundant. However, after the consumption of the initial Fe^2+^, growth rate became limited by the dissolution rate. After the first 24 h, the growth rate observed was μ_net_ = 0.017 ± 0.002 h^−1^, similar to the dissolution rate but 11-fold slower than the growth of this strain in its optimal conditions^23^. At the end of the culture, it was observed a 124-fold increase in cell concentration.

#### Sealed vial experiments without CO_2_

**Figure 3:**
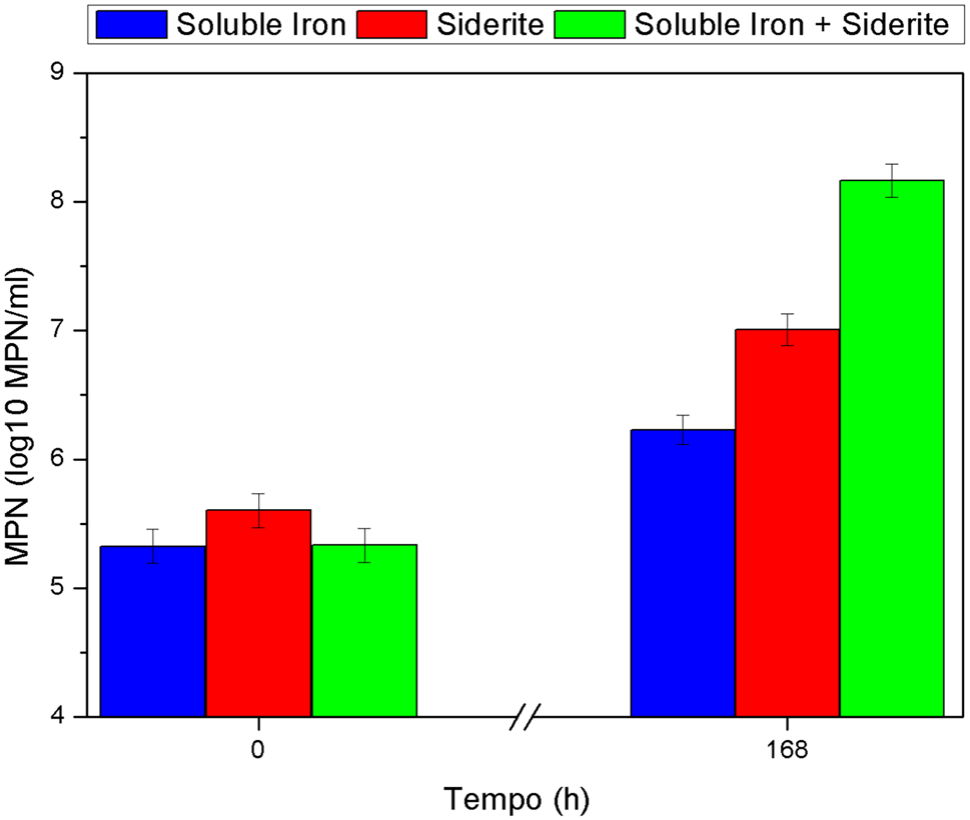
Determination of culturable cells (MPN) during the flask experiments without CO_2_ in the three conditions: presence of soluble iron (120 mM of FeSO_4_·H_2_O), presence of siderite (equivalent to ~ 5 mM of pure FeCO_3_), and presence of both together.

To probe the ability of the *A. ferrooxidans* to grow using siderite as its sole source of Fe^2+^ and carbon (assimilated as inorganic carbon by CO_2_ fixation), an experiment with a CO_2_-free atmosphere was designed. The assay was designed to run during one week to give enough time for the organism to grow, but after about three days the typical orange color of the high concentrations of Fe^3+^ was already observed in the biotic flasks with siderite-FeSO_4_ medium. This condition was the only one that also presented the deposition of minerals precipitated in the walls of the flask. Table 2 presents the concentrations of the Fe^2+^ and Fe^3+^ at the beginning and at the end of the experiment. The abiotic condition using just T&K medium showed a high level of abiotic oxidation of the Fe^2+^, producing 13.8 ± 1.6 mM of Fe^3+^. Meanwhile, the biotic condition reached an even higher Fe^2+^ oxidation, reaching a twofold higher concentration of Fe^3+^ at the end point. Yet, the Fe^2+^ available at the end was still higher than 100 mM in both cases. The fact that in the biotic condition showed a higher Fe^3^^+^ oxidation indicated that some of the iron oxidation was the biotic process of energy gain and that it may not be directly coupled to the carbon fixation for the biomass production. The abiotic condition of the siderite-FeSO_4_ medium showed a similar pattern of the abiotic condition using just T&K medium with a Fe^3+^ concentration of 16.8 ± 0.5 mM. However, the biotic counterpart showed a very different result, reaching 142.2 ± 13.4 mM of Fe^3+^ with only 0.3 ± 0.1 mM of Fe^2+^ available, of which nothing was found as soluble Fe^2+^ (not shown). As mentioned, precipitate deposition was seen over the walls of the flask and the final total iron recovery in the biotic condition was different from the abiotic condition recovery. The abiotic condition of the siderite medium also has some degree of oxidation and production of Fe^3+^ (3.2 ± 0.2 mM), yet just half of the produced in the biotic condition (6.9 ± 0.2 mM). An important difference between the two conditions was the accumulation of Fe^2+^ in solution in the abiotic (4.7 ± 0.1 mM), while the biotic culture showed just a minimal concentration of 0.29 ± 0.04 mM of Fe^2+^ in solution, a value similar to those seen during the bioreactor biotic cultures. Yet, not all siderite was solubilized, and it was still found at the bottom of the flask, probably due to the poor mixing when comparing the conditions found in the bioreactor. All the Fe^2+^ oxidation in the abiotic conditions can be explained by the presence of oxygen in the CO_2_-free synthetic air mixture used in the holdup gas exchange^23^. This helps to highlight the effect of the presence of molecular oxygen (O_2_)^52^ and other species on Mars that can lead to Fe^2^^+^ oxidation and the formation of abiotically induced minerals of astrobiological interest, like jarosite, in natural environments, making the process of identifying unambiguous biosignatures more complicated. The MPN assay results for the biotic conditions (Fig. 3) shows that in all three conditions tested the increase of the cell concentration was observed , though in very different scales: the T&K medium condition showed only a slight increase in cell concentration, up to eightfold the initial concentration,the siderite medium condition presented a 25-fold increase in cell concentration,and the siderite-FeSO_4_ medium showed the most expressive growth, up to 681-fold the initial concentration.

**Table 2:** Summary of the concentrations of Fe^2+^ and Fe^3+^ at the beginning and end of the flask experiments without CO_2_ in the six different conditions in triplicate.

Medium		Total Fe^2+^		Total Fe^3+^		Log_10_ MPN/ml	
		Initial	Final	Initial	Final	Initial	Final
T&K (FeSO_4_)	Abiotic	143.64 ± 0.76	132.3 ± 6.76	1.58 ± 0.06	13.86 ± 1.58	–	–
	Biotic	145.74 ± 2.14	118.7 ± 10.51	1.70 ± 0.03	26.37 ± 7.85	5.32 ± 0.00	6.32 ± 0.85
Siderite	Abiotic	5.48 ± 0.47	6.15 ± 0.39	2.12 ± 0.31	3.24 ± 0.23	–	–
	Biotic	4.98 ± 0.20	2.18 ± 0.46	2.24 ± 0.10	6.91 ± 0.20	5.61 ± 0.17	7.01 ± 0.27
Siderite-FeSO_4_	Abiotic	150.06 ± 5.08	148.60 ± 1.47	3.99 ± 0.11	16.82 ± 0.58	–	–
	Biotic	5.48 ± 4.53	0.33 ± 0.11^a^	3.54 ± 0.07	142.19 ± 13.42^a^	5.33 ± 0.12	8.17 ± 0.23

^a^Denote values with higher quantification error due to loss of material located on the flask wall.

#### Sealed vial experiments with inert gas

The experiment with an inert holdup gas was designed to access the ability of *A. ferrooxidans* to grow using siderite as its sole source of carbon (assimilated as inorganic carbon by CO_2_ fixation) in an environment without contact with the atmosphere (or contact with an inert atmosphere). The assay was carried out during one week to give enough time for the organism to grow while reducing the previously produced Fe^3+^ in a great extent, which can be observed as a color change from the typical strong orange to a light yellowish hue (still from the presence of Fe^3+^). All the 6 flasks had from the preparatory growth in T&K and before being sealed in the CO_2_-less conditions, 63.9 ± 0.4 mM of Fe^3+^ and a minimal residual concentration of Fe^2+^ lower than 1 mM. Both conditions, with and without siderite, showed a reduction of Fe^3+^ to Fe^2+^, as the reaction of sulfur oxidation with iron reduction to gain energy is not directly coupled to the carbon fixation for biomass production. Nevertheless, the presence of residual atmospheric gases cannot be completely ruled out. The condition without siderite showed a production of 15.2 ± 0.3 mM of Fe^2+^, leaving 33.6 ± 2.3 mM of Fe^3+^ in solution, pointing to the possible precipitation of some iron (probably as jarosite and/or schwertmannite, whose solubility in the medium pH is lower than the Fe^2+^ species), but that is visually indiscernible from the sulfur powder in suspesion (that is also insoluble). However, the condition with siderite showed a much higher reduction of iron, presenting 32.7 ± 6.4 mM of Fe^2+^ and only 15.9 ± 5.9 mM of Fe^3+^ in solution, that is, a concentration of reduced iron two-fold higher than the condition without siderite. The cell concentration with the added siderite went from 6.0 × 10^7^ MPN/mL (7.78 ± 0.17 log_10_MPN/mL) to 1.1 × 10^8^ MPN/mL (8.05 ± 0.09 log_10_MPN/mL), compared to a final value of 1.9 × 10^7^ MPN/mL (7.28 ± 0.11 log_10_MPN/mL) without siderite, demonstrating the growth of *A. ferrooxidans* only in the presence of this mineral.

### Vivianite—experiments with baffled Erlenmeyer flasks

**Figure 4:**
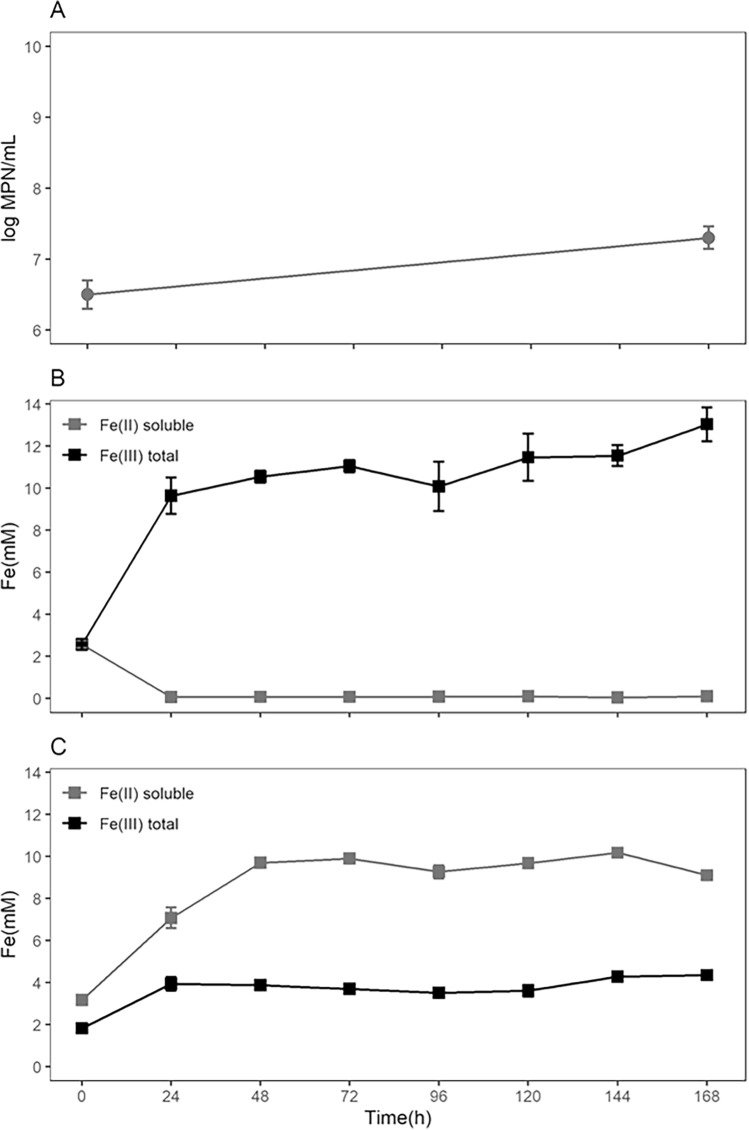
Flask experiments with baffled Erlenmeyer flasks using vivianite. (**a**) Determination of culturable cells (MPN) at the beginning and the end of the biotic experiments. (**b**) Biotic assay showing the organism mediated soluble Fe^2+^ oxidation and total Fe^3+^ accumulation. (**c**) Abiotic assay showing the vivianite dissolution with Fe^2+^ accumulation and small increase of total Fe^3+^ concentration (very low oxidation) compared to the original content in the mineral.

**Figure 5:**
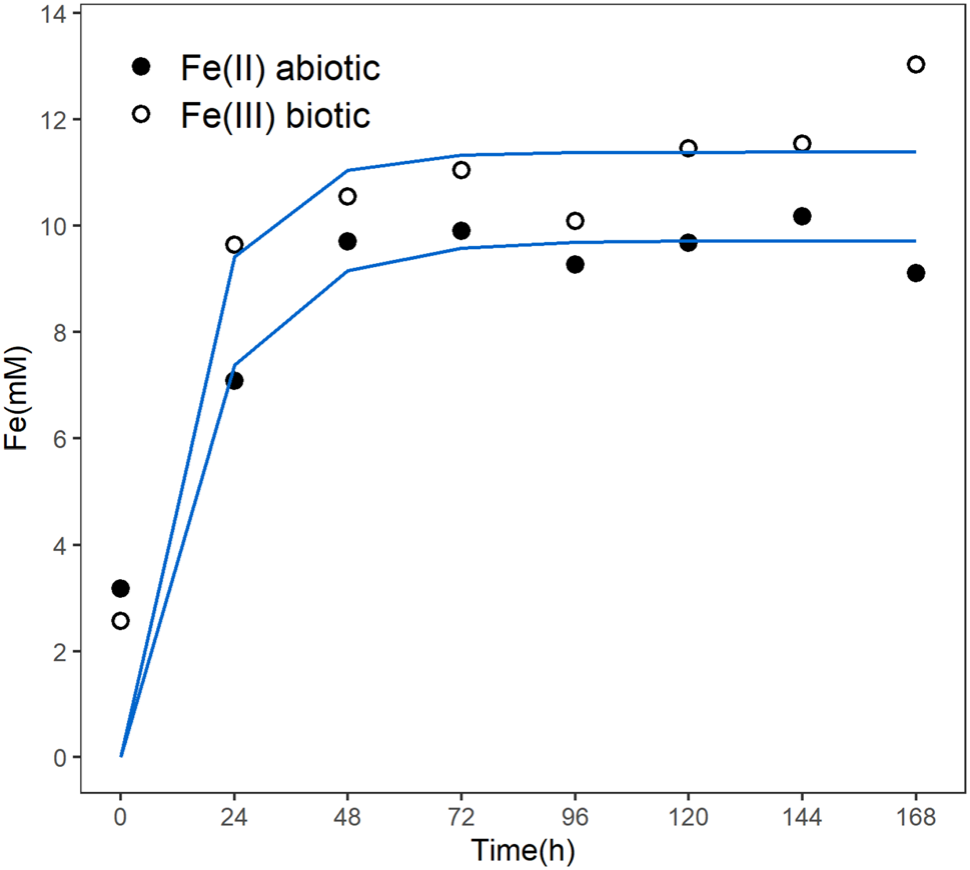
First-order model fitting for the vivianite dissolution during the flask experiments with baffled Erlenmeyer flasks experiments by the direct accumulation of Fe^2+^ (abiotic assay) and accumulation of Fe^3+^ after the biotic oxidation of Fe^2+^ to Fe^3+^ (biotic assay) (see Section “Modelling”).

The abiotic assay was performed to evaluate the dissolution rate of the vivianite in the culture medium and to quantify the natural oxidation rate of the Fe^2+^ ion without the presence of the microorganisms. However, differently from the bioreactor experiments using siderite, to perform the vivianite experiments baffled Erlenmeyer flasks were used, incubated in temperature- regulated shaker tables. This setup allows the control of all the main environmental conditions (30 °C and constant aeration), except for the pH. The abiotic medium was kept under these conditions during 168 h, during which the vivianite dissolution and the color change of the solution occurred (going from an opaque navy blue to a lighter but still opaque blue color). The mineral dissolution was fast and happened mainly in the first 48 h of the experiment, represented by the fast increase of the soluble Fe^2+^ concentration succeeded by a stationary phase that lasted until the end of the experiment (Fig. 4c). The total Fe^3+^ concentration in solution was 3.6 ± 0.7 mM at the end. Meanwhile, during the experiment, the ratio of the concentrations of total Fe^3+^/Fe^2+^ was in agreement with the Fe^3+^/Fe^2+^ ratio observed in the chemical formula obtained for the mineral, within a maximum error < 20% (not shown). The constancy in Fe^3+^ concentration and the similarity in the Fe^3+^/Fe^2+^ ratio during the experiment indicates that the main source of Fe^3+^ was the mineral per se, endorsing a condition of low abiotic oxidation. Again, the dissolution pattern was interpreted as similar to a kinetics of a first-order reaction and, after corrected by the ratio of Fe^2+^ in vivianite molecule (2.202:1), the fitting in such model gave a dissolution rate for vivianite of k = 0.027 ± 0.011 h^−1^ (Fig. 5), which, considering the specific surface area, represents 2.93*10^–9^ mol/(s*m^2^) of Fe^2+^.

The biotic assay was performed similarly to the abiotic assay, but with the addition of the stock cell suspension (2% v/v) from an *A. ferrooxidans* culture at the exponential growth phase. As previously stated, this introduces a small amount of Fe^2+^ and Fe^3+^ in the biotic experiment flasks from the previous culture and those values are subtracted during modelling the dissolution rate. Like in the biotic siderite experiments, it is shown that once the initial Fe^2+^ is oxidized by the *A. ferrooxidans* in the first 24 h, its concentration is kept low until the end at 168 h (Fig. 4b). The total Fe^3+^ concentration showed a fast increase during the first 24 h then kept some stability until the end of the experiment, in contrast to the abiotic assay where just after 48 h no substantial increase was observed. Despite the similarity of the total Fe^3+^ concentration curve in the biotic assay and the soluble Fe^2+^ concentration in the abiotic assay, it was observed an average difference of 5.1 ± 1.9 mM between them. This difference is compatible with the average total Fe^3+^ concentration in the abiotic assay, showing the conversion of the soluble Fe^2+^ in Fe^3+^ by *A. ferrooxidans*. Due to the lower volume of culture medium and the impossibility of online pH measurements in this apparatus, it was opted to not perform the pH control in this experiment and to perform the MPN methodology to determine the concentration of culturable cells only at the beginning and the end of the assay, providing only the overall growth of the *A. ferrooxidans*. A sixfold increase in cell concentration was verified in the vivianite experiments (Fig. 4a), however, the magnitude of this increment was much smaller than the observed for the bioreactor experiments using siderite. After subtracting the total Fe^3+^ present in the cultures that came from the addition of the vivianite (calculated from the abiotic assay) and correcting the molar ratio of Fe^2+^, it was possible to perform a fitting to estimate a dissolution rate for vivianite of k = 0.033 ± 0.014 h^−1^ (Fig. 5), which, considering the specific surface area, represents 3.85*10^–9^ mol/(s*m^2^) of Fe^2+^. Based o n the behavior observed for the siderite assay (Fig. 1b) of dissolution as a limiting factor and the concordance between MPN and the total Fe^3+^ curves, the direct value of the dissolution rate, without the molar correction of Fe^2+^_,_ is also interpreted as the value of the growth rate of the *A. ferrooxidans*, whose value was μ_net_ = 0.07 ± 0.03 h^−1^. A summary of the dissolution and growth rates can be found in Table 1. This growth rate is almost threefold slower than the rate of this strain in the optimal conditions^23^ and fourfold higher than the one estimated for the siderite assay. Contrary to the siderite experiments, it was noticed only a minimal amount of solid phase material (powdered vivianite and precipitated minerals) stuck on the flask walls in this experiment using baffled Erlenmeyer flasks. Reflecting the fast increase of the Fe^3+^ in the insoluble phase, it was observed that the medium went from an opaque navy blue to a lighter but still opaque yellowish-blue color.

## Discussion

### Siderite

The study of the habitability of environments beyond our planet is of great importance for understanding the possibility of the existence of past or present life outside the Earth and our ability to colonize those environments. Mars is today the focus of a space race and many missions have been and still are dedicated to studying and scrutinizing all aspects of its composition and chemical and geological conditions. This helps to bring a great influx of information and discoveries from different kinds of environments on the planet that could sustain or could have sustained life as we know it. Considering the actual conditions in the planet, the lack of organic compounds imply that an autotrophic metabolism could be advantageous for organisms living there^2^. Techniques employed today to explore the mineralogical composition of Mars only allow the identification of the major phases that make up the crust of the planet. Yet, some common minerals identified, like olivine, are able to sustain chemolithoautotrophic life from Earth^53^. The presence of siderite was already inferred in different places of Mars through analyses made by orbiters and rovers, and it was also detected in Martian meteorites recovered on Earth^31^. Although not accurately covering the simulation of the complete environmental conditions, the presence of these and other minerals allowed geochemical simulations of complex Martian regolith that demonstrated the capacity of minerals from the planet to sustain the development of chemolithoautotrophic organisms such as *A. ferrooxidans*^42^.

During the biotic experiments in the bioreactor in the presence of siderite, the biotic oxidation of the Fe^2+^ released by dissolution of the mineral and the fast increase in cell concentration of *A. ferrooxidans* demonstrated the capacity of this mineral to sustain the microbial growth, functioning as its sole source of energy. Yet, the similarity in the growth rate of the organism and the dissolution rate of the mineral implied that the dissolution process can be a limiting factor for the growth. The slower dissolution of the mineral in the biotic assay shows that the microorganism can, somehow, influence in this process. As mentioned before, o ne hypothesis proposed is the larger production of EPS in the presence of a solid substrate^48^ that could cover the mineral, slowing its dissolution. However, the reason for this slowdown is not yet known, and it was not possible to assess the existence of any direct action of the microorganism towards the decrease of the mineral dissolution rate. This comes in contrast with the known process of EPS-mediated dissolution carried by this microorganism on sulfide minerals^54^.

Large amounts of superficial water are not present on Mars, however subsurface lakes were already detected in the Southern Hemisphere of the planet, although their exact environmental conditions (like temperature and salinity) are not yet known^4^. This detection brings more elements to the debate about the presence of liquid water in the planet and if the conditions necessary for that are still found there nowadays. For swallow areas, the very low concentration of oxygen could be able to directly sustain an aerobic metabolism^42^, while simulations show that other aqueous near-surface environments could also be supplied by dissolved oxygen diffusing through supercooled brines in contact to the atmosphere in sufficient quantities to sustain the aerobic life, specially near the poles^55^. The exact salt compositions of such brines are unknown, however the presence of perchlorates^56^, sulfates^6^ and carbonates^31^ are implied due to their detection in high concentrations in the planet. *A. ferrooxidans* is very susceptible to the presence of chlorides and perchlorates, however can survive in high concentrations of MgSO_4_, up to 10% (w/v)^28^. Cullen et al.^51^ have shown that siderite can dissolve in acidic brine conditions even in the presence of high concentrations of MgSO_4_, up to 3 mol/L. Thus, this indicates that a sufficiently warm environment on Mars with the presence of siderite in acidic brines of MgSO_4_ with atmospheric supply of oxygen could bear the presence of a chemolithoautotrophic organism like *A. ferrooxidans*.

The experiments without CO_2_ and with an inert atmosphere demonstrate that siderite can also contribute to the habitability of environments devoid of contact with the atmospheric CO_2_. In these assays, the final cell concentration increase was not the same as the bioreactor assay, probably due to the far-from-ideal mixing condition of the flasks in the shaker table, leading to an incomplete dissolution of the siderite, and the lack of control of the pH of the solution. On the other hand, the cell concentration of the cultures showed a small, but statistically relevant growth. These conditions can blur the evidence of the capability of siderite to be a carbon provider, however, in the experiments without CO_2_, the medium enriched with soluble Fe^2+^ (siderite-FeSO_4_ medium) showed that the siderite can not only be the sole energy source for *A. ferrooxidans*, but also can provide the inorganic carbon to its production of biomass. This finding effectively expands the conditions known to be capable to support life as we know. Meanwhile, while both conditions with inert gas showed Fe^3+^ reduction, the statistically relevant difference between the conditions with and without siderite also reinforces its ability as an inorganic carbon provider for *A. ferrooxidans* to produce its biomass. In a broader view, siderite could also contribute to the habitability of past Mars as the carbonate deposits are associated with water weathering of rocks bearing Mg and Fe minerals in an atmosphere of high partial and absolute CO_2_ pressure^31^. During the complex history of Mars, it was possible that this siderite could then be exposed to acidic environments^57^ and oxidizing conditions that suggest the presence of oxygen^58^. As said before, the putative presence or absence of CO_2_ in subsurface lakes should not interfere in its growth potential when siderite, or other carbonate minerals, is present. Nevertheless, the presence of aqueous environments in the surface of Mars presents an alternative niche in which the presence of life can be assessed. One of the challenges for life to thrive on Mars today, and probably also in the past, is the high levels of radiation flux experienced by an organism living on its surface^59^, being that *A. ferrooxidans* is especially sensitive to UVC^28^. On Earth, *A. ferrooxidans* can be found adhered to the surface of minerals, if a similar situation happened on Mars, a thin layer of oxidized iron from the regolith could provide a protection to UV radiation^18^. However, in the proposed aqueous scenario, t he fast dissolution of the siderite and the biotic oxidation of Fe^2+^, together with the iron from the regolith, could be the providers of Fe^3+^, known to absorb large amounts of UVC, creating a protective layer even in low concentrations or in shallow waters^28,60^. Meanwhile, despite the fact that no sulfur (S_8_) has been directly detected on Mars yet, the past of the planet points to strong volcanic activity and elemental sulfur associated to that might have been present^61^. Again, subsurface lakes on Mars could provide minerals to sustain a microorganism like *A. ferrooxidans* even if there is no contribution of gases from the atmosphere or locally generated oxygen, through anaerobic growth and mineral carbon fixation. This way, the association of different conditions with simple minerals and species like sulfur, siderite and soluble Fe^3+^ could expand the environments on Mars that could be habitable to life as we know it.

### Vivianite

Among the main biogenic elements (usually referred to by the mnemonic CHNOPS), phosphorus is the one with the lowest concentration in the universe and can be considered a concern during the search for habitable environments^62^. However, some estimations about the concentration of this element on Mars point to the possibility of mantellic concentrations up to tenfold higher than that on Earth^38^. The main phosphates found in meteorites can be readily leached in acidic water, while some possible secondary phosphates formed from this process, like Fe-phosphates, also show rapid dissolution rates^63^. The solubilization and precipitation cycle that could arise from this in a planet with abundant acidic aqueous environments can contribute to a high mobility of phosphorus in the planet, reflecting in the formation of patches of soil with up to 2.4% wt. of P^64^. Nevertheless, the exact minerals that bear this phosphorus are yet unknown, and a high number of candidates appears in a list of phosphates whose signal is indistinguishable by the equipments employed in the exploration of the planet (for example^38^. During the growth experiments, the mineral chosen was vivianite, a relatively common Fe^2+^-bearing phosphate found on Earth and whose presence on Mars is considered a possibility . As seen during the growth experiments using siderite, the biotic oxidation of Fe^2+^ when compared to the abiotic assays and the significant increment in the cell concentration showed that *A. ferrooxidans* is able to use vivianite as its sole source of energy. Likewise to the siderite, the vivianite could be present in superficial and subsuperficial acidic lakes in past and current subsuperficial brines on Mars, expanding the number of known Fe^2+^ sources able to provide energy to organisms with metabolism similar to that of *A. ferrooxidans*. Besides being a source of rapidly- dissolved Fe^2+^, vivianite could also provide to the aqueous environment a great influx of bioavailable phosphate, an important resource for life. The similarity of the concentration curve of soluble Fe^2+^ and the Fe^3+^ produced by the microorganisms (total Fe^3+^ minus the Fe^3+^ contained in the mineral) show that the limiting factor during the *A. ferrooxidans* growth is the dissolution of the mineral and that it is not possible to assess the existence of any direct action of the microorganism towards the increase or decrease of the mineral dissolution. Different from the siderite experiments, a significant difference was not seen on the solubilization of the mineral between biotic and abiotic assays. Considering the hypothesis of EPS production influencing the mineral solubilization, a faster dissolution of vivianite in the biotic assays (threefold faster than the siderite) may not have left the necessary time for cells to produce enough EPS to affect the dissolution rate. This could be corroborated by the absence of precipitate deposition over the flask walls, associated with a more turbulent flux introduced by the baffled Erlenmeyer flasks. On the other hand, the unavailability of pH control prevented the maintenance of the cultures in the ideal value of ~ 1.8, leading to an underestimation of the vivianite dissolution rate. Although the exact values of the pH during the experiments were not obtained, the formation of ferric oxides and inhibition of the *A. ferrooxidans* at pH > 2.5 can draw a maximum value for the solution pH during biotic and abiotic experiments^65^. This could also help to explain the poor results of cellular growth. The influx of Fe^2+^ to solution in the vivianite biotic assay was almost sevenfold higher than the influx during the siderite biotic assay, while the growth rate was only fourfold higher. Yet, the imprecision of indirect measure of the growth rate during the vivianite experiments could also introduce higher uncertainties.

## Conclusion

The intense investigation of the surface of Mars has been revealing a complex geological history entangled in the extreme environmental changes that the planet suffered. Associated to these processes, there is the emergence of many major and minor mineral phases, some already identified and some yet to be uncovered. Those minerals may still hide an unexplored potential to sustain microorganisms, providing essential elements for the growth of chemolithoautotrophic communities. Thus, it was shown that Fe^2+^ from siderite and vivianite can be accessed by the acidophilic bacterium *Acidithiobacillus ferrooxidans*, so that the ion oxidation is the only energy source for the development of the organism. In addition, siderite can provide both the iron species for energy and the carbon, in the form of the carbonate ion, for the cells to produce their own biomass. Thus, the only external input necessary for an aerobic, iron-oxidizing growth of the bacterium in siderite is a small amount of oxygen or, in an anaerobic gasless condition, the coupling of sulfur oxidation and Fe^3+^ reduction with the carbon fixation from siderite. The occurrence of these conditions might have been common in ancient Mars and isolated subsurface environments that share some of these characteristics could still be plausible in the present. The growth of microorganisms using the minerals as described expands the list of possible scenarios able to harbor the elements necessary for the flourishing of life outside Earth. Therefore, these novel geomicrobiological processes should be taken into account during the analysis of an extraterrestrial environment, broadening its astrobiological potential and prospects of its habitability.

### Supplementary Information

Supplementary Information 1.

## Data Availability

The datasets generated during and/or analyz ed during the current study are not publicly available but are available from the corresponding author on request.

## References

[1] Grotzinger J, Beaty D, Dromart G, Gupta S, Harris M, Hurowitz J, Kocurek G, McLennan S, Milliken R, Ori GG, Sumner D (2011). Mars sedimentary geology: Key concepts and outstanding questions. Astrobiology.

[2] Nicholson WL, Rampelotto P (2018). Experimental evolution to explore adaptation of terrestrial bacteria to the martian environment. Molecular Mechanisms of Microbial Evolution (Grand Challenges in Biology and Biotechnology).

[3] Cockell CS (2020). Astrobiology: Understanding Life in the Universe.

[4] Lauro SE, Pettinelli E, Caprarelli G, Guallini L, Rossi AP, Mattei E, Cosciotti B, Cicchetti A, Soldovieri F, Cartacci M, Di Paolo F, Noschese R, Orosei R (2021). Multiple subglacial water bodies below the south pole of mars unveiled by new MARSIS data. Nat. Astron..

[5] Dartnell LR (2011). Ionizing radiation and life. Astrobiology.

[6] Chevrier V, Mathé PE (2006). Mineralogy and evolution of the surface of Mars: A review. Planet Space Sci..

[7] Salese F, Pondrelli M, Neeseman A, Schimidt G, Ori GG (2019). Geological evidence of planet-wide groundwater system on mars. J. Geophys. Res. Planet.

[8] Chevrier V, Poulet F, Bibring JP (2007). Early geochemical environment of Mars as determined from thermodynamics of phyllosilicates. Nature.

[9] Eigenbrode JL, Summons RE, Steele A, Freissinet C, Millan M, Navarro-Gonzáles R, Sutter B, McAdam AC, Franz HB, Glavin DP, Archer PD, Mahaffy PR, Conrad PG, Hurowitz JA, Grotzinger JP, Gupta S, Ming DW, Sumner DY, Szopa C, Malespin C, Buch A, Coll P (2018). Organic matter preserved in 3-billion-year-old mudstones at Gale crater, Mars. Science.

[10] Hurowitz JA, Grotzinger JP, Fischer WW, McLennan SM, Milliken RE, Stein N, Vasavada AR, Blake DF, Dehouch E, Eigenbrode JL, Fairén AG, Frydenvang J, Gellert R, Grant JA, Gupta S, Herkenhoff KE, Ming DW, Rampe EB, Schmidt ME, Seibach KL, Stack-Morgan K, Sumner DY, Wiens RC (2017). Redox stratification of an ancient lake in Gale crater. Mars. Sci..

[11] Neilson JW, Quade J, Ortiz M, Nelson WM, Legatzki A, Tian F, LaComb M, Betancourt JL, Wing RA, Soderlund CA, Maier RM (2012). Life at the hyperarid margin: Novel bacterial diversity in arid soils of the Atacama Desert, Chile. Extremophiles.

[12] De Sá JS, Mezzomo H, Fraga MF, Ogrodowski CS, Santana FB (2017). Anode air exposure during microbial fuel cell operation inoculates with maarine sediment. J. Environ. Chem. Eng..

[13] Pathak A, Morrison L, Healy MG (2017). Catalytic potential of selected metal ions for bioleaching, and potencial techno-economic and environmental issues: A critical review. Bioresourc. Technol..

[14] Cockell CS (2010). Geomicrobiology beyond Earth: microbe-mineral interactions in space exploration and settlement. Trends Microbiol..

[15] Cockell CS, Santomartino R, Finster K, Waajen AC, Eades LJ, Moeller R, Rettberg P, Fuchs FM, Van Houdt R, Leys N, Coninx I, Hatton J, Parmitano L, Krause J, Koehler A, Caplin N, Zuijderduijn L, Mariani A, Pellari SS, Carubia F, Luciani G, Balsamo M, Zolesi V, Nicholson N, Loudon C-M, Doswald-Winkler J, Herová M, Rattenbacher B, Wasdworth J, Everroad RC, Demets R (2020). Space station biomining experiment demonstrates rare earth element extraction in microgravity and Mars gravity. Nat. Commun..

[16] Loudon C, Nicholson N, Finster K, Leys N, Byloos B, Van Houdt R, Rettberg P, Moeller R, Fuchs FM, Dements R, Krause J, Vukich M, Mariani A, Cockell C (2018). BioRock: New experiments and hardware to investigate microbe–mineral interactions in space. Int. J. Astrobiol..

[17] Krot AN, Keil K, Scott ERD, Goodrich CA, Weisberg MK, Holland HD, Turekian KK (2014). Classification of meteorites and their genetic relationships. Treatise on Geochemistry.

[18] Gómez F, Mateo-Martí E, Prieto-Ballesteros O, Martín-Gago J, Amils R (2010). Protection of chemolithoautotrophic bacteria exposed to simulated Mars environmental conditions. Icarus.

[19] Gonzáles-Toril E, Martínez-Frías J, Gómez JMG, Rull F, Amils R (2005). Iron meteorites can support the growth of acidophilic chemolithoautotrophic microorganisms. Astrobiology.

[20] Gronstal A, Pearson V, Kappler A, Dooris C, Anand M, Poitrasson F, Kee TP, Cockell CS (2009). Laboratory experiments on the weathering of iron meteorites and carbonaceous chondritees by iron-oxidizing bacteria. Meteorit. Planet Sci..

[21] Kölbl D, Blaevic A, Albu M, Fasching C, Milojevic T (2020). Desiccation of the extreme thermoacidophile metallosphera sedula grown on terrestrial and extraterrestrial materials. Front. Astron Space Sci..

[22] Milojevic T, Albu M, Kölbl D, Kothleitner G, Bruner R, Morgan ML (2021). Chemolithotrophy on the Noachian Martian breccia NWA 7034 via experimental microbial biotransformation. Nat. Commun. Earth Environ..

[23] Gonçalves Silva G, Yamassaki de Almeida E, Seber P, Henrique Settanni P, Pereira de Oliveira A, Ferreira Santos MS, Lucio do Lago C, Cieslarova Z, Rodrigues F, (2018). Application of capillary electrophoresis combined with conductometric and UV detection to monitor meteorite simulant bioleaching by Acidithiobacillus ferrooxidans. Electrophoresis.

[24] Valdés J, Pedroso I, Quatrini R, Dodson RJ, Tettelin H, Blake R, Eisen JA, Holmes DS (2008). Acidithiobacillus ferrooxidans metabolism: from genome sequence to industrial applications. BMC Genom..

[25] Caraballo MA, Rimstidt JD, Macías F, Nieto JM, Hochella MF (2013). Metastability, nanocrystallinity and pseudo-solid solution effects on the understanding of schwertmannite solubility. Chem. Geol..

[26] Nazari B, Jorjani E, Hani H, Manafi Z, Riahi A (2014). Formation of jarosite and its effect on important ions for acidithiobacillus ferrooxidans bacteria. Trans. Nonferr. Metal Soc..

[27] Hynek BM, McCollom TM, Szynkiewwicz A (2019). Sulfur cycling and mass balance at Meridiani, Mars. Geophys. Res. Lett..

[28] Bauermeister, A. Characterization of stress tolerance and metabolic capabilities of acidophilic iron-sulfur-transforming bacteria and their relevance to mars. Dissertation, Universität Duisburg-Essen (2012)

[29] Sutter B, Boynton WV, Ming DW, Niles PB, Morris RV, Golden DC, Lauer HV, Fellows C, Hamara DK, Mertzman SA (2012). The detection of carbonate in the martian soil at the Phoenix Landing site: A laboratory investigation and comparison with the Thermal and Evolved Gas Analyzer (TEGA) data. Icarus.

[30] Bridges JC, Catling DC, Saxton JM, Swindle TD, Lyon IC, Grady MM (2001). Alteration assemblages in martian meteorites: implications for near-surface processes. Space Sci. Rev..

[31] Bridges JC, Hicks LJ, Treiman AH, Filiberto J, Schwenzer SP (2019). Carbonates on mars. Volatiles in the Martian Crust.

[32] Zavarzina DG, Kochetkova TV, Chistyakova NI, Gracheva MA, Antonova AV, Merkel AY, Perevalova AA, Chernov MS, Koksharov YA, Bonch-Osmolovskaya EA, Gavrilov SN, Bychkov AY (2020). Siderite-based anaerobic iron cycle driven by autotrophic thermophilic microbial consortium. Sci. Rep..

[33] Köhler I, Konhauser KO, Papineau D, Bekker A, Kappler A (2013). Biological carbon precursor to diagenetic siderite with spherical structures in iron formations. Nat. Commun..

[34] Vuillemin A, Wirth R, Kemnitz H, Schleicher AM, Friese A, Bauer KW, Simister R, Nomosatryo S, Ordoñez L, Ariztegui D, Henny C, Crowe SA, Benning LG, Kallmeyer J, Russell JM, Bijaksana S, Vogel H, the Towuti Drilling Project Science Team (2019). Formation of diagenetic siderite in modern ferruginous sediments. Geology.

[35] Rothe M, Kleeberg A, Hupfer M (2016). The occurrence, identification and environmental relevance of vivianite in waterlogged soils and aquatic sediments. Earth-Sci. Rev..

[36] Miot J, Benzerara K, Morin G, Kappler A, Bernard S, Obst M, Férard C, Skouri-Panet F, Guigner JM, Posth N, Galvez M, Brown GE, Guyot F (2009). Iron biomineralization by anaerobic neutrophilic iron-oxidizing bacteria. Geochim Cosmochim Ac.

[37] Kappler A, Newman DK (2004). Formation of Fe(III)-minerals by Fe(II)-oxidizing photoautotrophic bacteria. Geochim Cosmochim Ac.

[38] Dyar DM, Jawin ER, Breves E, Marchand G, Nelms M, Lane MD, Mertzman SA, Bish DL, Bishop JL (2014). Mössbauer parameters of iron in phosphate minerals: Implications for interpretation of martian data. Am. Min..

[39] Garcia O (1991). Isolation and purification of Thiobacillus ferrooxidans and Thiobacillus thiooxidans from some coal and uranium mines of Brazil. Rev. Microbiol..

[40] Tuovinen OH, Kelly DP (1973). Studies on the growth of Thiobacillus ferrooxidans. Arch. Mikrobiol..

[41] Mendham, J., Denney, R. C., Barnes, J. D. & Thomas, M. J. K. Vogel’s Quantitative Chemical Analysis, Vol. 6th edition. 780 Pearson Education, (London, 2000).

[42] Bauermeister A, Rettberg P, Flemming HC (2014). Growth of the acidophilic iron–sulfur bacterium Acidithiobacillus ferrooxidans under Mars-like geochemical conditions. Planet Space Sci..

[43] Jarvis B, Wilrich C, Wilrich PT (2010). Reconsideration of the derivation of most probable numbers, their standard deviations, confidence bounds and rarity values. J. Appl. Microbiol..

[44] Vusikhis AS, Gulyaeva RI, Leont'ev LI, Ovchinnikova LA, Selivanov EN (2016). Kinetic features of breunnerite decarbonization. Russ. Metall..

[45] Chukanov NK, Scholz R, Aksenov SM, Rastsvetaeva RK, Pekov IV, Belakovskiy DI, Krambrock K, Paniago RM, Roghi A, Martins RF, Belotti FM, Bermanec V (2012). Metavivianite, Fe^2+^Fe^3+^_2_(PO_4_)_2_(OH)_2_·6H_2_O: new data and formula revision. Mineral Mag..

[46] Lozano-Calero D, Martín-Palomeque P, Madueño-Loriguillo S (1996). Determination of phosphorus in cola drinks. J. Chem. Educ..

[47] Luna-Zaragoza D, Romero-Guzmán ET, Reyes-Gutiérrez LR (2009). Surface and physicochemical characterization of phosphates vivianite, Fe_2_(PO_4_)_3_ and hydroxyapatite, Ca_5_(PO_4_)_3_OH. J. Min. Mat. Charact. Eng..

[48] Gehrke T, Telegdi J, Thierry D, Sand W (1998). Importance of extracellular polymeric substances from thiobacillus ferrooxidans for bioleaching. Appl. Environ. Microb..

[49] Levenspiel O (1999). Chemical reaction engineering.

[50] Shuler ML, Kargi F, DeLisa M (2017). Bioprocess Engineering: Basic Concepts.

[51] Cullen MD, Phillips-Lander CM, Elwood Madden ME (2017) Siderite Dissolition Kinetics in Mars-Analog Brines. Lunar and Planetary Science XLVIII. https://www.hou.usra.edu/meetings/lpsc2017/pdf/2379.pdf 2379. Accessed 15 July 2022.

[52] Moroz VI (1998). Chemical composition of the atmosphere of mars. Adv. Space Res..

[53] Popa R, Smith AR, Popa R, Boone J, Fisk M (2012). Olivine-respiring bacteria isolated from the rock-ice interface in a lava-tube cave, a mars analog environment. Astrobiology.

[54] Harneit K, Göksel A, Kock D, Klock JH, Gehrke T, Sand W (2006). Adhesion to metal sulfide surfaces by cells of Acidithiobacillus ferrooxidans, Acidithiobacillus thiooxidans and Leptospirillum ferrooxidans. Hydrometallurgy.

[55] Stamenković V, Ward LM, Mischna M, Fischer WW (2018). O_2_ solubility in Martian near-surface environments and implications for aerobic life. Nat. Geosci..

[56] Hecht MH, Kounaves SP, Quinn RC, West SJ, Young SMM, Ming DW, Catling DC, Clark BC, Boynton WV, Hoffman J, DeFlores LP, Gospodinova K, Kapit J, Smith PH (2009). Detection of perchlorate and the soluble chemistry of Martian soil at the phoenix lander site. Science.

[57] Peretyazhko TS, Niles PB, Sutter B, Morris RV, Agresti DG, Le L, Ming DW (2018). Smectite formation in the presence of sulfuric acid: Implications for acidic smectite formation on early Mars. Geochim Cosmochim Ac.

[58] Noda N, Imamura S, Sekine Y, Kurisu M, Fukushi K, Terada N, Uesugi S, Numako C, Takahashi Y, Hartmann J (2019). Highly oxidizing aqueous environments on early mars inferred from scavenging pattern of trace metals on manganese oxides. J. Geophys. Res. Planet.

[59] Cockell CS, Catling DC, Davis WL, Snook K, Kepner RL, Lee P, McKay CP (2000). The ultraviolet environment of mars: Biological implications past, present, and future. Icarus.

[60] Gómez F, Aguilera A, Amils R (2007). Soluble ferric iron as an effective protective agent against UV radiation: Implications for early life. Icarus.

[61] Franz HB, King PL, Gaillard F, Filiberto J, Schwenzer SP (2019). Sulfur on Mars from the atmosphere to the core. Volatiles in the Martian Crust.

[62] Márcia-Barber E (2020). The Chemical Evolution of Phosphorus: an Interdisciplinary Approach to Astrobiology.

[63] Tu VM, Hausrath EM, Tschauner O, Iota V, Egeland GW (2014). Dissolution rates of amorphous Al- and Fe-phosphates and their relevance to phosphate mobility on Mars. Am Min..

[64] Gellert R, Rieder R, Brückner J, Clark BC, Dreibu G, Klingelhöfer G, Lugmair G, Ming DW, Wänke H, Yem A, Zipfel J, Squyres SW (2006). Alpha particle X-ray spectrometer (APXS): Results from Gusev crater and calibration report. J. Geophys. Res. Planet.

[65] Meruane G, Vargas T (2003). Bacterial oxidation of ferrous iron by Acidithiobacillus ferrooxidans in the pH range 2.5–7.0. Hydrometallurgy.

