# Supplementary figures and images for: Siderite and vivianite as energy sources for the extreme acidophilic bacterium Acidithiobacillus ferrooxidans in the context of mars habitability
Source: Sci Rep. 2024 Jun 27;14:14885. doi: 10.1038/s41598-024-64246-7 (PMC11211326; doi:10.1038/s41598-024-64246-7)

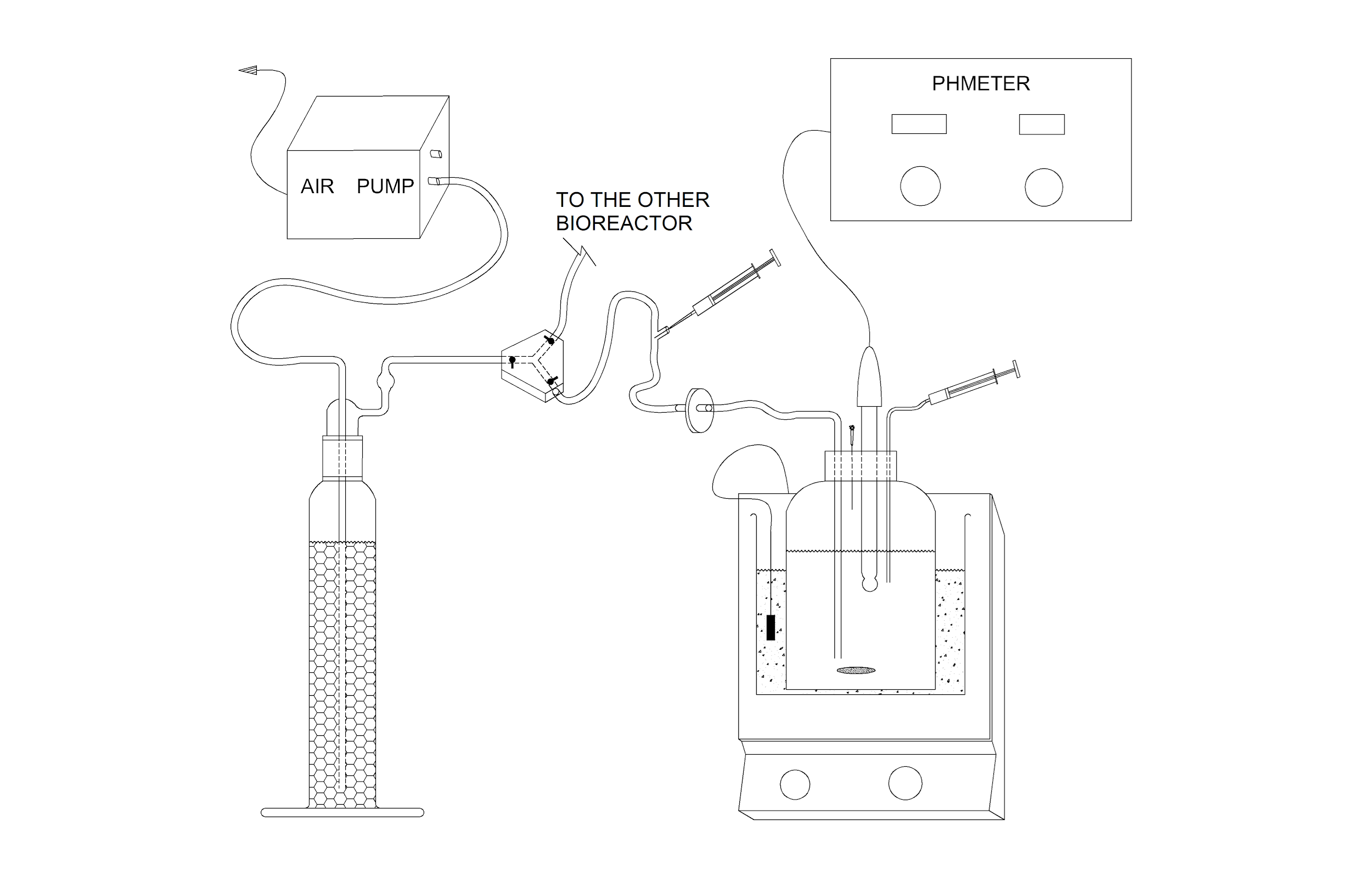

Supplement: Supplementary file 1 — Supplementary Information 1. [file 41598_2024_64246_MOESM1_ESM.tif]
